# Improving youth mental wellness services in an Indigenous context in Ulukhaktok, Northwest Territories: ACCESS Open Minds Project

**DOI:** 10.1111/eip.12816

**Published:** 2019-06-27

**Authors:** Meghan Etter, Annie Goose, Margot Nossal, Jessica Chishom‐Nelson, Carly Heck, Ridha Joober, Patricia Boksa, Shalini Lal, Jai L. Shah, Neil Andersson, Srividya N. Iyer, Ashok Malla

**Affiliations:** ^1^ ACCESS Open Minds Ulukhaktok Ulukhaktok Northwest Territories Canada; ^2^ Inuvialuit Regional Corporation Inuvik Northwest Territories Canada; ^3^ ACCESS Open Minds (Pan‐Canadian Youth Mental Health Services Research Network) Douglas Mental Health University Institute Montreal Quebec Canada; ^4^ Department of Nursing McGill University Montreal Quebec Canada; ^5^ Department of Psychiatry McGill University Montreal Quebec Canada; ^6^ Prevention and Early Intervention Program for Psychosis (PEPP) Douglas Mental Health University Institute Montreal Quebec Canada; ^7^ School of Rehabilitation, Faculty of Medicine Université de Montréal Montreal Quebec Canada; ^8^ Centre de recherche du Centre hospitalier de l'Université de Montréal (CRCHUM) Montreal Quebec Canada; ^9^ Department of Family Medicine, Community Information and Epidemiological Technologies (CIET) Institute and Participatory Research at McGill (PRAM) McGill University Montreal Quebec Canada; ^10^ McGill Institute of Human Development and Well‐being McGill University Montreal Quebec Canada

**Keywords:** culture, Indigenous, Inuit, lay health worker, youth mental health, Canada

## Abstract

**Aim:**

To describe a community‐specific and culturally coherent approach to youth mental health services in a small and remote northern Indigenous community in Canada's Northwest Territories, under the framework of ACCESS Open Minds (ACCESS OM), a pan‐Canadian youth mental health research and evaluation network.

**Methods:**

As 1 of the 14 Canadian communities participating in a 5‐year, federally funded service transformation and evaluation project, the arctic Inuit community of Ulukhaktok has undertaken culturally relevant adjustments in their delivery of youth mental wellness services and related community wellness initiatives. These enhancement activities highlight connections to culture and traditional skills, honour youth‐ and community‐expressed desires to incorporate Inuvialuit‐specific approaches to wellness, and strengthen the support systems to improve access to mainstream mental healthcare, when needed. The adaptation of a Lay Health Worker model from Global Mental Health to the local circumstances resulting in creation of lay community health workers is central to this approach in meeting contextual needs.

**Results:**

Community leaders identified key activities for sustainable change, including human capital development, authentic collaboration and diversified engagement strategies. Building around five ACCESS OM objectives, the local site team in Ulukhaktok has identified its youth programming and mental wellness service gaps through an ongoing process of community mapping.

**Conclusions:**

Information from service providers, youth and other community members demonstrates attuning of the ACCESS OM framework to Inuit paradigms in Ulukhaktok. It could prove to be a sustainable prototype for delivering youth mental health services in other communities in the Inuvialuit Settlement Region and possibly across the entire Inuit Nunangat. It needs, however, to be further supported by easier access to specialized mental health services when needed.

## 1 INTRODUCTION

Addressing mental health needs of young people should be a priority for healthcare in Canada (Malla, Shah, et al., 2018). Frequently reported high rates of adverse mental health and suicide in Indigenous communities are of particularly grave concern (Boothroyd, Kirmayer, Spreng, Malus, & Hodgins, 2001; Health Canada, 2013). The urgency of this is accentuated by the fact that Indigenous communities have relatively high proportions of population under the age of 25 (40%‐50%) (Turner, Crompton, & Langlois, 2011). Risk factors, related to the worst consequences of colonialism and the large‐scale intergenerational trauma, are associated with high rates of mental health problems and addiction while also negatively influencing acceptance of non‐Indigenous services (Nelson & Wilson, 2017).

In response to growing concerns about grossly inadequate mental health services available to youth living in very diverse geographic, cultural and historical circumstances in Canada, a pan‐Canadian service transformation project, ACCESS Open Minds (ACCESS OM) was launched in 2014. ACCESS OM, initiated under the auspices of the Strategies for Patient‐Oriented Research (SPOR), comprises 14 very diverse sites, including four First Nations and two Inuit communities, as outlined in a recent report on ACCESS OM (Malla, Srividya et al., 2018).

Here, we describe how this service is being transformed in the remotely situated Inuit community of Ulukhaktok. The preparatory phase of the ACCESS OM project, launched in Ulukhaktok in September 2016, involved a feast attended by approximately one‐quarter of the community. The site team was fully staffed by early 2017 and activities have been underway since. An official launch of the ACCESS OM Youth Space is anticipated for 2019.

## 2 BACKGROUND

Based on the key principles and objectives of the national project (Malla, Srividya et al., 2018), the ACCESS OM implementation in Ulukhaktok is built by local community members within the context of unique characteristics of the community, the people, and their lived reality as 21st century Ulukhaktokmuit, or people of Ulukhaktok.

### 2.1 Geographical context

Inuit have thrived in the high arctic for more than a millennium, harvesting food from the land and the icy waters, and passing on tradition and culture through spoken word, art and practice. Connection to the land, culture and history is woven into the fabric of an Inuvialuit understanding of wellness, and by extension to the understanding of mental health.

Ulukhaktok is a small hamlet of 396 people (Statistics Canada, 2017) in Canada's western arctic, part of the Inuvialuit Settlement Region (ISR) on the western coast of Victoria Island. Almost all community residents have knowledge of English. While the traditional language, Inuinnaqtun, is the mother tongue for 130, an additional 85 have knowledge of it. Until “settlements” in the 1930s were established under colonial rule, Inuit were traditionally nomadic, having set up communities throughout the region following the seasons for their traditional practices of hunting, fishing and gathering food from the land.

Regularly accessible only by air, Ulukhaktok is geographically remote; the tundra landscape, above the tree‐line, is rocky and for much of the year covered in snow and ice. Apart from access by air, an annual barge from the south makes larger deliveries (building supplies, dry goods, or vehicles). The community hosts the world's northernmost golf tournament and has also hosted international cruise ships navigating the Arctic Ocean's Northwest Passage.

### 2.2 Socio‐historical context

The history of Inuvialuit and their experience of colonization is directly relevant for any discussion of the provision of services, especially mental health services in Ulukhaktok. Among the principal sources of intergenerational trauma is the effect of residential schools. Indigenous children from this community, as elsewhere in Canada, were forcibly removed from their families as early as five years old and transported to residential schools, under the Indian Act (Truth and Reconciliation Commission of Canada, 2015). The latter enforced compulsory attendance for all children in order to assimilate Indigenous children into “mainstream” Canada and to ultimately “kill the Indian in the child” (De Leeuw, 2009). Punished for speaking their language, prohibited from practising their culture, and made to believe that being Indigenous was “sinful,” these children suffered high rates of malnutrition and emotional, physical and sexual abuse (Truth and Reconciliation Commission of Canada, 2015). In the Inuvialuit Settlement Region, the last school was closed as late as 1996.

## 3 STATUS OF MENTAL HEALTH SERVICE PROVISION

As a first step towards service transformation contextualized to local realities, within ACCESS OM each site engaged in an exercise of “community mapping” in order to understand the current status of service delivery within each community.

### 3.1 Community mapping results

Established in 1984, the Inuvialuit Regional Corporation (IRC) represents the collective Inuvialuit interests in dealings with governments. IRC is responsible for “continually improving the economic, social and cultural well‐being of all Inuvialuit,” (Inuvialuit Regional Corporation, 2018) including ACCESS OM.

Initiated by an external consultant, IRC took charge of the ACCESS OM project, its strategies, management and site‐level activities in partnership with Ulukhaktok Community Corporation. Mental health services are incorporated within general health services in that the counsellor is located in the community health centre. Non‐Indigenous nurses from the south provided general health services to youth, travelling in and out of the community for 8 weeks at a time. One staff member lived in the community for part of the year job‐sharing with another nurse. Other staff (social worker, childcare worker) also visited the community from time to time. Members of the community often hold the positions of Community Health Representative, Home Support Worker and Student and Family Support Worker.

The small geographical hamlet promotes a central location for the provision of multiple services. These are located in the School, Health Centre, Kayutak Centre and the Hamlet Office. A Youth Centre, situated adjacent to the Community Centre and the school, serves youth from all elementary and secondary grades and hosts an after‐school recreation program, including, sports, games and computer access. The Community Centre provides culturally coherent activities such as drum dancing, cooking programs and community feasts. Older youth (18‐25 years), however, often do not engage in the pre‐existing activities. In addition to healthcare providers, other service providers such as teachers, the school principal and the Royal Canadian Mounted Police (RCMP; the federal police agency serving Ulukhaktok) are often preferred points of contact for youth with mental health problems, presumably, due to reluctance to reveal such concerns to those who are known to them as fellow community members.

### 3.2 Service deficiencies

During planning stages of the ACCESS OM project, the community team identified a lack of mental health knowledge or local skills in this community to provide support to young community members. They wanted their own knowledge and skills to deal with young people when they have mental health difficulties, and to use their traditional land‐ and sea‐based activities to support them. There are no psychiatric services available locally, and youth in severe crises (e.g., psychosis, severe substance abuse, suicidal attempt or overdose) are referred to hospitals further south, minimum of one‐and‐a‐half‐hour flight away from the community. The ACCESS OM local community team wanted the skills to provide aftercare to young people on their return from these hospital stays, to identify young people with mental health and addiction problems, and to be able to support them locally.

Apart from difficulties envisaged in hiring a full‐time ACCESS Clinician (Malla, Srividya et al., 2018), it was highly unlikely that such a staff position would necessarily be in the best interest of youth. Professional staff would ordinarily deliver “standardized” mental health services. In this setting, however, the local community team felt that service providers needed better understanding of local youth and a trusting relationship and, therefore, considerable investment in and connection to the community. The team expressed that there may be lingering mistrust of “mainstream” mental health services and general stigma towards mental health. As an alternative, they proposed that a local resident—someone with in‐depth understanding of the community and its unique needs—would be better able to fill the gap, their exact role redefined for the context.

### 3.3 Creating a mental health service based on local needs and resources

Based on deficiencies identified by the local Inuit community, especially a lack of culturally appropriate and relevant professional resources, and the remote location of the community, an innovative alternate model of care seemed appropriate. Such a model of care must be feasible, based on some degree of prior evidence and the community's highest priorities. The national ACCESS OM leadership team had previous experience of providing training to local lay health workers (LHWs) in shifting key tasks from professionals to the trained LHWs in a low‐resource rural setting (Malla et al., 2019). Tasks safely shifted to trained LHWs include identifying individuals with emerging or persistent mental health problems and substance abuse, support in seeking additional professional help and to those who received professional interventions elsewhere.

In Ulukhaktok, community mapping clarified these issues. One impact of multigenerational trauma is the active struggle in this community to keep a strong connection with culture while increasing ownership of resources, including healthcare. In some other parts of the world where there has been widespread trauma and violence, communities have introduced alternate models of providing mental health services (Chan, Parish, & Yellowlees, 2015; Humayun, Azad, Khan, Ahmad, & Farooq, 2016; Malla et al., In press; Mendenhall et al., 2014; Rathod et al., 2017; Weissbecker, Hanna, El Shazly, Gao, & Ventevogel, 2019).

### 3.4 Adapting and applying a LHW model of care to Ulukhaktok

The success of implementing a LHW model depends on: careful local adaptation to prevailing geographic, cultural and economic conditions; adequate training focused on the specific role LHWs take on and tasks they will perform; cultural and linguistic congruity with those in need of the services; external professional back‐up and resources to deal with mental health problems that are beyond the capacity of LHWs; and adequate availability and appropriate use of technology (Chan et al., 2015; Hilty et al., 2013; Hubley, Lynch, Schneck, Thomas, & Shore, 2016; Moirangthem et al., 2017).

The Ulukhaktok team decided that this approach could be adapted adequately to the local circumstances, as a non‐threatening, culturally relevant option for youth seeking mental health supports and services. The team adapted LHW role and training for an *ACCESS OM Youth Worker* (AYW). Two AYWs recruited from the local community (initially one young person and one elder) received training which was a combination of training derived from World Health Organization (WHO) training material and Indigenous‐focused modules. The latter included Mental Health First Aid‐Inuit, Applied Suicide Intervention Skills Training (ASIST), CPR/First Aid, bullying education through the Red Cross and an adaptation of the WHO mhGAP intervention guide was provided by ACCESS OM central office.

Given the unique cultural context, the training was conducted through a series of conversations between the AYWs in the community and the training staff at the ACCESS OM central office, wherein the material from the WHO Lay Health Worker model (document) was presented. Subsequent discussions that ensued involved how such an concept might fit into the Inuvialuit way of thinking and how the AYWs might see this concept being applicable to them in their community. In particular, we used the wisdom and experience of one of the two initial AYWs, an Elder from the community. The latter has significant experience being a support person/helper and working in her community's school and as a teacher of traditional ways. As such, the discussions were generally facilitated to amplify her voice in learning how such concepts around community mental health work (as written from a more global perspective) might apply to the incorporation of the ACCESS OM framework in Ulukhaktok.

Now integral to the community site team, these workers focus on outreach activities, provide education about mental health issues, guide youth to appropriate services, and work collaboratively with youth, service providers, and other community members to provide better mental health care for youth. The Ulukhaktok team considered the best way to connect with all youth is to build strong connections with the AYWs and with other supportive adults in the community. AYWs facilitate youth events and activities, including fishing trips, cooking workshops, arts and crafts projects, and other on‐the‐land programming (e.g., hunting, igloo building, collaborations with other initiatives such as Project Jewel, an on‐the‐land wellness program through IRC).

## 4 MEETING THE FIVE OBJECTIVES OF THE ACCESS OM FRAMEWORK IN ULUKHAKTOK

The ACCESS OM framework is built around five foundational objectives to be addressed at service sites in diverse ways due to the vast differences in cultural context, geography and population (Malla, Srividya et al., 2018). This framework allows for a community's contextual differences to be the strength of their service delivery. How the ACCESS OM objectives are being achieved in Ulukhaktok is detailed below.

### 4.1 Early identification

The first objective of the ACCESS OM project is to identify more youth who are experiencing a mental health problem sooner in order to reduce untreated prevalence of mental health problems. The approach of estimating untreated prevalence based on census data from Statistics Canada and the Canadian Community Health Survey (CCHS) is used for non‐Indigenous sites but is inappropriate for most Indigenous communities due to the lack of their inclusion in the CCHS. Further, Inuit might not share the same cultural concept of mental disorders as non‐Inuit or non‐Indigenous peoples of Canada. The community team recognizes, however, an urgent need to improve general mental health literacy and to identify young people with emerging mental health concerns. Working closely with schools and other agencies (through community mapping), the AYWs are able to discuss issues related to mental health and substance abuse. Recently, collaboration between the local Royal Canadian Mounted Police and youth leaders resulted in an information session within the school on common mental health issues, online resources and telephone help lines. Engagement and identification of school‐aged youth has been one of the site team's strengths; the school administration often shares their concerns regarding individual students with the AYWs. The engagement activities, initiated by the AYWs, bring youth together and facilitate the learning of cultural practices. This allows them to connect with the youth in their community in a way that no outside‐sourced professional could.

### 4.2 Rapid access

The second objective of ACCESS OM is that youth in need (or those acting on their behalf) are offered an initial evaluation of their presenting problem within 72 hours of seeking help. In Ulukhaktok, this objective is modified since the AYWs do not provide mental health assessments, especially if the problem appears to be severe. The AYWs connect with help‐seeking young people quickly and guide them and their families towards the most appropriate services. In general, rapid access to *someone* in the regular services is rarely a concern in Ulukhaktok due to the small size of the community. Rapid access to specialized mental health interventions, such as addictions treatment or psychiatric care, however, requires leaving the community. This can take significant time to arrange and, at the same time, may not be adequate or appropriate. The AYWs' role includes helping navigate these pathways towards care and supporting youth and their family members in the journey, in the quickest way possible. The AYWs are now in the process of obtaining training to be able to offer support to youth as they return to the community after seeking care elsewhere.

### 4.3 Appropriate care

The ACCESS OM framework intends to connect youth to high quality, needs based, evidence‐informed, culturally appropriate and youth‐friendly care within 30 days (Malla, Srividya et al., 2018). For the ACCESS OM Ulukhaktok team, “appropriate care” means several things including that interventions are aligned with the cultural context. AYWs can provide general mental health and personal support, including land‐based and culturally appropriate programming. But specific treatment for more severe mental disorders like psychosis, suicidal crisis and severe addiction is not currently available locally. Use of technology (eg, tele‐psychiatry) to facilitate access to care will be explored in future.

### 4.4 Continuity of care

Within the ACCESS OM framework, any transition in care should be based on clinical need and not chronological age (Malla, Srividya et al., 2018; Osgood, Foster, & Courtney, 2010; Singh et al., 2010; Singh & Tuomainen, 2015). In the case of Ulukhaktok, as in many other Indigenous communities, there is no real division between these two age‐based systems of care. The division of child‐adolescent and young adult services remains problematic when youth require psychiatric care outside the community in non‐Indigenous settings. By building relationships with the mental healthcare system outside the community, the AYWs hope to better support young people (and their families) who have left the community for care, and to better manage the transition once they return.

### 4.5 Youth and family engagement

As a project funded under the Canadian Institutes of Health Research (CIHR)'s Strategy for Patient‐Oriented Research, engagement of youth and families is fundamental to service transformation under the ACCESS OM project. In Ulukhaktok, engaging family members and carers in ACCESS OM initiatives has been challenging despite sharing information through both formal (e.g., presentations) and informal social networks (e.g., word‐of‐mouth, online presence). Many community members, young and old alike, describe a marked generational divide between youth and Elders, but also a desire for increased intergenerational connectivity in the community. As well, over the course of the first two years of ACCESS OM activities, parents and young families have been identified as in need of further exposure to learning about mental health. There is also some hesitation expressed by youth in Ulukhaktok to share their mental health concerns, such as suicidal thoughts, with their own families. The AYWs have started hosting “family nights” at the community hall and are planning other activities, such as support groups for parents to engage older adults in discussions of mental health.

### 4.6 Research and evaluation

A fundamental component of the ACCESS OM project is to evaluate each objective of the service transformation through collection of individual, as well as program level data, using an evaluation protocol (Iyer, Jordan, MacDonald, Joober, & Malla, 2015). As the transformation proceeded (described above), it also became obvious that the standard measures used at other sites were not possible to apply with the same precision and expectations in this small Inuvialuit community of less than 400 people. Apart from the small numbers expected to meet criteria for a mental health disorder, the lack of tradition of recording responses in written format (as per the originally stated protocol) was an important facet of the site. Therefore, in consultation with community leadership, data will be collected mostly in the form of stories and personal narratives using a qualitative research strategy, as well as some preliminary interviews conducted by a trained graduate nursing student who met with the youth informally. The details of the qualitative strategy to be applied to all sites will be discussed in a separate report. Follow‐up community mapping activities comparing the pre‐ and post‐implementation overview of services and activities in the community and reports of skill‐building, on‐the‐land wellness programs, both in the community and beyond, will form part of the evaluation.

## 5 DISCUSSION

In this report, we have described how the ACCESS OM service transformation was adapted to the very special circumstances of a small arctic (Inuit) community, with particular attention to the unique cultural context, effects of intergenerational trauma related to colonialism, the deliberate suppression of their nomadic culture, variable and largely inadequate mental health resources for provision of appropriate care and absence of local ownership of mental health resources. This cultural attuning of the ACCESS OM framework includes training two local Inuvialuit AYWs to support youth in the community in social, cultural and land‐based activities. We expect this will assist early identification of emerging and existing mental health problems. Their role also involves connecting youth in need to professional services available in the community from the non‐Indigenous, territorial‐funded system, supporting more satisfactory transitions to seeking services outside the community, and supporting youth when they return from episodes of care outside of the community.

### 5.1 Challenges

The community reports that older youth (18‐25) are less likely to engage in basic support services and participate in culturally appropriate activities compared to the younger youth. Some of the former are working, starting families and leaving the community to pursue post‐secondary education. In response to the recurrent request by youth to create more opportunities to connect to their culture and learn about mental wellness, the transmission of traditional knowledge became central to ACCESS OM activities in Ulukhaktok. Older youth have requested advanced Inuinnaqtun instructions, sewing and beading classes, and lengthier on‐the‐land camps. Facilities are currently being built to create a separate youth centre, attached to the sports arena, where they will have access to activities and supportive resources in collaboration with the AYWs.

Being central to the service transformation locally, AYWs must manage a dual identity. Being members of the small remote community, they have their own identities as parents, siblings, community members and, in the case of an Elder AYW, grandparent and respected Elder. Seen as trusted confidants by the youth, the AYWs also have to identify youth with mental health problems who may need additional professional help while such access is often not easy. They have to live up to expectations of being trusted community members and at the same time navigate issues of confidentiality between the youth, their families, and those in positions of perceived authority (e.g., school administrators, child and family workers and police).

Given the small size and close‐knit nature of the community, difficulty in maintaining privacy when seeking help was seen by youth as a challenge and by AYWs as a barrier to early identification of mental health problems. Youth viewed receiving help for mental health as a threat to social acceptance by both peers and family. Youth also state that parents and carers do not generally ask in‐depth questions about mental wellness. They feel they are expected to rely on themselves to cope with threats to their mental health. The most common alternatives to reaching out for clinical support included disclosing to a trusted friend or engaging in cultural practices such as being on the land (Ulukhaktok youth, personal communication, July 6 & 19, 2018).

The challenge of meeting the ACCESS OM benchmarks for rapid access is grounded in the fact that no specialized care, such as addiction or psychiatric interventions, are available within Ulukhaktok; this care requires travel outside the community, which also has an impact upon continuity of care.

## 6 CONCLUSION

Much of the care being provided by the local ACCESS OM team is not directed at specific mental health interventions that would treat individual problems, but rather the team is helping to build supportive and integrated community responses to improve overall mental health literacy and wellness. Future efforts should also support the development of structures and use of technology that can help youth presenting with identifiable mental health and addictions crises, or who might require specialized mental healthcare. Collaborative work with the site team and their regional partners is not only a solution for amplifying their voices in this research project, but also in how this community response to improving mental health services could shape youth mental healthcare in other remote, arctic locations across Canada's north. Stigma and confidentiality remain challenges that the local team continues to work on to find creative solutions, such as increasing activity‐based interventions for older youth to be started in the new venue.
